# Variations in *BCO2* Coding Sequence Causing a Difference in Carotenoid Concentration in the Skin of Chinese Indigenous Chicken

**DOI:** 10.3390/genes14030671

**Published:** 2023-03-08

**Authors:** Yan Wang, Shiyi Gan, Chenglong Luo, Sijia Liu, Jie Ma, Wei Luo, Chuxiao Lin, Dingming Shu, Hao Qu

**Affiliations:** 1 State Key Laboratory of Livestock and Poultry Breeding, Guangdong Key Laboratory of Animal Breeding and Nutrition, Institute of Animal Science, Guangdong Academy of Agricultural Sciences, Guangzhou 510640, China; 2 Guangdong Polytechnic of Science and Trade, Guangzhou 510430, China

**Keywords:** yellow skin, expression profile, β-carotene oxygenase 2, carotenoid

## Abstract

**Simple Summary:**

The deposition of carotenoids in chicken skin makes the skin color turn from white into yellow. The enzyme β-carotene oxygenase 2 (BCO2) plays a key role during the degradation process of carotenoids in chicken skin. Hence, the concentration of carotenoids in chicken skin was measured, and significant differences in *BCO2* gene expression in the back skin between yellow and white skin and one SNP c.890A>G in *BCO2* were found to be potentially associated with the chicken skin color. The results of this study showed that the c.890A>G may be used as a genetic marker in breeding for yellow skin in Chinese indigenous chicken.

**Abstract:**

Carotenoid consumption decreases the risk of cancer, osteoporosis, or neurodegenerative diseases through interrupting the formation of free radicals. The deposition of carotenoids in chicken skin makes the skin color turn from white into yellow. The enzyme β-carotene oxygenase 2 (BCO2) plays a key role during the degradation process of carotenoids in skin. How the *BCO2* affects the skin color of the chicken and whether it is the key factor that results in the phenotypic difference between yellow- and white-skin chickens are still unclear. In this research, the measurement of the concentration of carotenoids in chicken skin by HPLC showed that the carotenoid concentration in chickens with a yellow skin was significantly higher than that in white-skin chickens. Moreover, there were significant differences in *BCO2* gene expression in the back skin between yellow- and white-skin chickens. Scanning the SNPs in *BCO2* gene revealed a G/A mutation in exon 6 of the *BCO2* gene in white and yellow skin chicken. Generally, one SNP c.890A>G was found to be associated with the chicken skin color and may be used as a genetic marker in breeding for yellow skin in Chinese indigenous chickens.

## 1. Introduction

Carotenoids are natural pigments that are synthesized by plants, algae, and some bacteria and fungi and are an important source of vitamin A for livestock [1]. In vivo, besides acting as pigments, carotenoids also have other biology functions; they can, for instance, function as antioxidants that have an important role in protecting DNA from oxidative damage [2]. Oxidative stress has been recognized as the main contributor to the age-related diseases such as atherosclerosis, osteoporosis, obesity, dementia, diabetes, cancer, and arthritis [3,4,5]. Carotenoid consumption is associated with a lower risk to develop these diseases by interrupting the propagation of free radicals [6,7,8]. Animals, however, cannot synthesize carotenoids de novo; they intake carotenoid from their food, with a way of absorbing in intestine and transporting in plasma exclusively by lipoproteins to various organs, such as skin. [9,10]. Carotenoids are therefore an important component in a well-balanced human diet. Similarly, carotenoids are included in the feed of production animals in a species-specific way to support animal health and achieve a high performance [11,12]. 

In poultry, feed high in carotenoid content leads to carotenoid-pigmented egg yolk, skin, legs, beak, comb, and feathers of some breeds. The skin color of chickens in some countries, such as China, is an important trait, influencing the choice of consumers in their decision to buy a chicken or not [13]. The skin color of chickens may differ depending on how much of the carotenoid pigment is deposited in the skin. Consumers think that skin pigmentation is an important health indicator in poultry, as weak health greatly reduces the absorption of carotenoids; thus, poultry farmers add carotenoids to chicken feed to achieve the desired color to satisfy customers’ preference. For the marketing of poultry products, in a number of countries in the world, chicken skin color is a trait that influences consumer behavior. A bright yellow skin color is often associated with freshness and health and has become an indicator of high-quality products. A uniform and good pigmentation generally means good health and good practical hygienic conditions [14]. In chickens, the capacity to accumulate carotenoids in skin is, however, mostly genetically determined [15]. The accumulation of carotenoids gives the skin a yellow color, while the enzymatic degradation of carotenoids or the absence of carotenoid absorption gives the skin a white appearance.

There are two enzymes involved in carotenoid metabolism, namely β-carotene 15, 15′-monooxygenase 1 (BCMO1) and β-carotene oxygenase 2 (BCO2). BCMO1 is responsible for the oxidative cleavage of β-carotene into two retinal molecules [4,5], while BCO2 can mediate the degradation of carotenoid into colorless substances [6,7,8,9]. The expression of these enzymes is tissue dependent. Recently, research has demonstrated that BCOM1 and BCO2 work together in the gut and liver in carotenoid degradation, while other tissues, e.g., adipose tissue, depend on BCO2 for carotenoid metabolism [9,16]. 

Several previous studies have revealed that point mutations in the *BCO2* gene can influence the deposition of carotenoids in the skin, thus influencing skin color. For example, a nonsense mutation (c.196C>T) leaving the *BCO2* gene inactive is associated with the accumulation of carotenoids in adipose tissue in sheep [9]. Furthermore, mutations in *BCO2* have been shown to affect the concentration of carotenoids in bovine milk [17]. In chickens, three SNPs, namely G>A, A>G, and G>A (chr24: 6,264,085 bp, 6,273,428 bp and 6,287,900 bp, WUGSC 2.1/galGal3), in the *BCO2* gene are associated with a yellow skin trait [15]. We also tested those SNPs in five Chinese indigenous chicken breeds, and the results showed that some breeds carried alleles at this locus that are not so closely related to white-skin or yellow-skin alleles in our domestic breeds. Some research studies have shown that the genetic structure is different between Western breeds and Chinese local chicken breeds; by whole-genome re-sequencing 126 chicken individuals, combined with the genome sequencing data of 31 chickens that were previously published, Luo et al. observed the highest levels of genome-wide genetic differentiation between each commercial population (White Recessive Rock chicken, Ross308 chicken, Rhode Island Red chicken, and White Leghorn chicken) and Chinese indigenous chickens, especially between White Leghorn chickens and Huiyang Bearded chickens [18,19,20]. The *BCO2* and its flanking genes (IL18 and PTS) are also consistent as candidate positively selected genes for the yellow pigmentation phenotype by the analysis of genome-wide scans for signals of selection [21]. 

Above all, *BCO2* is a strong candidate gene to affect the deposition of carotenoids. The aim of this investigation was to study differences in *BCO2* expression between yellow-skin and white-skin chickens. This will help us understand the molecular mechanisms involved in skin carotenoid metabolism and provide a valuable theoretical basis for the selection of the skin trait during the selective breeding of Chinese indigenous chickens. 

## 2. Materials and Methods 

### 2.1. Ethics Statement

Our study was approved by the Animal Care Committee of the Institute of Animal Science, Guangdong Academy of Agricultural Sciences (Guangzhou, China), with approval number GAAS-IAS-2009-73. All birds were housed in individual battery cages with ad libitum access to food and water and humanely euthanized.

### 2.2. Samples

Two breeds of chicken were used. The Guangxi Huang (S4, with yellow skin) chickens were bred by the Institute of Animal Science, Guangdong Academy of Agricultural Sciences (Guangzhou, China). The Qingjiao Ma (Q, with white skin) chickens were bred by the Sichuan Agricultural University (Ya’an, China), which kindly provided the samples used in this study. All birds had free access to feed and water, used the same feed, and no additional carotenoids were added to the feed. 

Screening for polymorphisms in chicken *BCO2* gene was performed in a total of 60 birds of the Guangxi Huang and Qingjiao Ma chickens, 30 birds in each breed. A total of 1054 DNA samples from eight different chicken breeds collected at the Institute of Animal Science, Guangdong Academy of Agricultural Sciences, were genotyped and determined allele frequencies: Huiyang Beard, Mahuang with black shank, Mahuang with navy shank, Youxi Ma, Qingjiao Ma, Fast-Growing Lingnanhuang Line A, and Guangxi Huang Chicken. These breeds or lines have all been kept by us or the Sichuan Agricultural University (Ya’an, China) for over 15 years (inbred for at least 15 generations), without interbreeding with any other breeds/lines.

At the age of 70 days, blood samples were collected in 1.5 mL tubes with 1.5% EDTA, stored at −20 °C, and then used for DNA isolation. Birds were humanely euthanized, and tissue samples, including heart, liver, spleen, lungs, kidney, muscular stomach, glandular stomach, intestine, breast muscle, ovary, cerebrum, cerebellum, hypothalamus, hypophysis, and back skin, were collected. Tissue samples were collected for RNA isolation and were put in 1.5 mL tubes with RNA later (Sigma-Aldrich, St. Louis, MO, USA) and stored at −20 °C. Genomic DNA was isolated from blood samples by using standard phenol and phenol/chloroform-purification-based protocols.

### 2.3. Measurement of Carotenoid Concentration in Chicken Skin

The measurement of the carotenoid concentration in chicken skin at the age of 70 days was performed in a total of 6 females of the Guangxi Huang and Qingjiao Ma chickens, 3 birds in each breed. Back skin tissue (0.5 g) was added to 5 mL mixture reagent (CHCL_3_:CH_3_OH = 2:1) in a 50 mL tube and homogenized. Then 5 mL mixture reagent was added to the homogenate, followed by 2 mL 0.9% (8.5 g/L) NaCl, after which the tube was vortexed for 2 min. Samples were centrifuge at 648 g for 10 min at 4 °C. After centrifugation, the CHCL_3_ layer was transferred to a new tube, while the water layer was transferred to another tube with 5 mL hexane, vortexed for 2 min, and centrifuged again at 648 g for 10 min at 4 °C. From this tube, the hexane layer was removed and added to the tube with the CHCL_3_ phase [22,23]. The content of the tube was put in a water bath at 50 °C and was dried by nitrogen, using a Rotary Evaporator (RE-3000A, Shanghai Yarong Biochemistry Instrument factory, Shanghai, China); the formed pellet was dissolved in 0.15~5 mL ethanol, after which 50 μL of the ethanol samples was analyzed using High-Performance Liquid Chromatography (LC-20AD, SHIMAZU Inc., Kyoto, Japan). Lutein and zeaxanthin were used as the internal standard (Guangzhou Juyuan Biochemical Co., Guangzhou, China) and measured at a wavelength of 445 nm and 451 nm, respectively. Differential carotenoid concentrations in chicken skin between the Guangxi Huang and Qingjiao Ma breeds were determined using a *t*-test with SAS 8.0 software (SAS Institute, Cary, NC, USA).

### 2.4. Primers

Primer pairs were designed for 18 fragments (F1/R1-F18/R18), together responsible for amplification of the complete CDS of the *BCO2* gene. Primers for PCR-RFLP: YSD-F/R, primers for RT-PCR: BCO2-F/R, and the housekeeping genes GAPDH-F/R and β-actin-F/R. Three primers, namely BCO2-A-F/R, BCO2-B-F/R, and BCO2-C-F/R, were also designed to identify the SNPs (SNP A, SNP B and SNP C), which were from before research [15]. All primers were synthesized by Sangon Biotech (Shanghai, China) Co., Ltd., Shanghai, China. The primer sequences are shown in Table 1.

### 2.5. SNP Scanning and Genotyping

The 18 fragments were amplified by PCR, using DNA as the template. To check whether the correct products were amplified, the PCR products were sequenced (Sangon Biotech (Shanghai) Co., Shanghai, China). PCR reaction amplification was performed in 25 μL reaction volume on a GeneAmp PCR system 9600 (Perkin Elmer, Foster City, CA, USA). Mixes comprised 100 ng chicken genomic DNA as a template, 1 μM of each primer, 1× PCR reaction mix, and 1 U Taq DNA polymerase (Dongsheng, Guangzhou, China). The PCR amplifications were performed with the following cycling parameters: initial denaturalization at 94 °C for 4 min, 35 cycles of 94 °C for 45 s, annealing at optimal temperature for 45 s, and 72 °C for 1 min. A final extension was performed at 72 °C for 10 min. All amplified products were separated on agarose gels and purified using a Gel Extraction Kit (Sangon). The purified PCR products were cloned into the pMD19-T vector (Takara) and sequenced in both directions. Potential polymorphic sites were analyzed by sequence comparison, using DNAstar software (DNAstar Inc., Madison, WI, USA). 

The sequences were aligned between the Guangxi Huang and Qingjiao Ma chickens to identify the SNP site. A 444 bp fragment was amplified using specific primer pairs (YSD-F/R, Table 1). For genotyping by PCR-RFLP assays, 7 μL of PCR products were digested with 4 U *Sdu*I (ThermoScientific, Shanghai, China) in a 1 × digestion buffer, in a total volume of 10 μL, following digestion for 6 h at 37 °C. Then the digested products were separated by electrophoresis on a 2.0% agarose gels. Three SNPs (SNP A, SNP B, and SNP C) were identified by PCR; we used primers BCO2-A-F/R, BCO2-B-F/R, and BCO2-C-F/R; and the PCR products were sequenced (Sangon Biotech (Shanghai) Co., Shanghai, China). 

### 2.6. Real-Time PCR

Three female chickens, 70 days old, of the Guangxi Huang and Qingjiao Ma breed (respectively) were used for this analysis. Fifteen tissues, namely heart, liver, spleen, lungs, kidney, gizzard (muscle part stomach), glandular stomach, intestine, breast muscle, ovary, cerebrum, cerebellum, hypothalamus, hypophysis, and back skin, were collected. The back skin tissues from three female chickens of Guangxi Huang and Qingjiao Ma breed, respectively, at 2 d, 21 d, and 42 d were also collected and transferred into RNAlater solution (Life Technologies, Carlsbad, CA, USA) and stored at −20 °C. Tissues were weighed (approximately 0.2 g of tissue was used), grounded into a powder in liquid nitrogen, and homogenized in 2 mL of TRIzol (Life Technologies, Rockville, MD USA), using a handheld electric homogenate instrument (Germany, Staufen, IKA). The homogenized samples were left for 5 min at room temperature and then centrifuged at 12,000 rpm (representative g value) at 4 °C for 10 min. Total RNA was isolated using the RNAiso Plus kit (Takara Bio Inc., Dalian, China) according to the manufacturer’s instructions. The amount and quality of the samples was estimated using the NanoDrop ND-1000 spectrophotometer (ThermoScientific, MA, USA) and agarose gel electrophoresis (Bio-Rad Laboratories, SYSTEM GelDoc XR+, Hercules, CA, USA). First-strand cDNA synthesis and reverse transcriptase PCR were performed as described in the instructions for the PrimeScript^TM^ II reagent Kit with gDNA Eraser (TaKara, Dalian, China). Primer pairs BCO2-F/BCO2-R (Table 1) were used to determine the relative expression values of the *BCO2* gene by qPCR. The PCR amplifications were performed in 20 μL reaction volumes comprising 0.5 μL of chicken cDNA, 0.5 μL of each primer, and 10 μL of SYBR Green Real-time PCR Master Mix (TOYOBO, Tokyo, Japan) in a LightCycler 480 Real-Time PCR System (Roche Applied Science, Indianapolis, IN, USA). The PCR conditions were 95 °C for 1 min, followed by 40 cycles of 95 °C for 15 s, 58 °C for 15 s, and 72 °C for 20 s. The level of fluorescence was used to calculate the threshold cycle (Ct) value for each sample. The relative gene-expression levels were analyzed using the comparative Ct method, in which the housekeeping genes *β-actin* and *GAPDH* (Table 1) were used as internal controls, and the geometric averaging of those two internal control genes was calculated according to a previously published method [24]. The geometric mean of 2 reference genes according to the ΔCT [16] 2^−ΔCT^ method were used to calculate the expression level [25]. Expression abundances of the *BCO2* gene in 15 tissue and back-skin samples from four different times, between the Guangxi Huang and Qingjiao Ma breeds, were determined and analyzed using a *t*-test with SAS 8.0 software (SAS Institute, Cary, NC, USA); differences showing *p* < 0.05 were considered significant.

## 3. Results

### 3.1. Carotenoid Concentration in Chicken Skin and Difference of BCO2 Expression Level in Tissues

**Figure 1:**
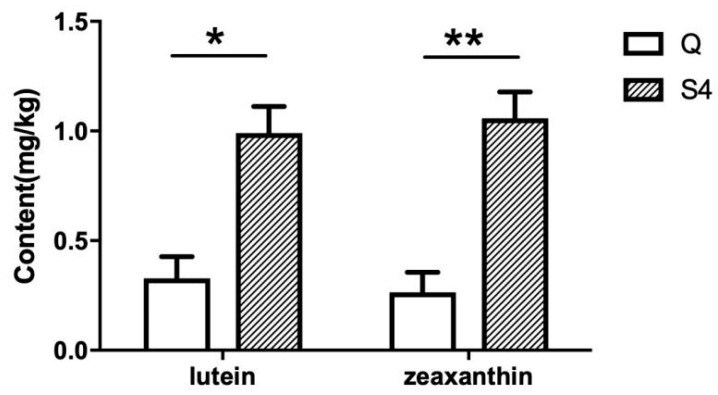
Concentrations of lutein and zeaxanthin in skin from chickens differing in their skin color by the HPLC. Data are expressed with means ± SE (*n* = 3). Q represents the Qing jiao ma as white-skin chicken, and S4 represents the Guangxi Huang as yellow-skin chicken; * and ** indicate significant differences between different breeds (* *p* ≤ 0.05; ** *p* ≤ 0.01).

The carotenoid concentration in chicken skin was determined by HPLC. No β-carotene was detected, but the lutein and zeaxanthin content of yellow-skin chicken-S4 were significantly (*p* < 0.05) higher than that of white-skin chicken-Q (Figure 1). 

**Figure 2:**
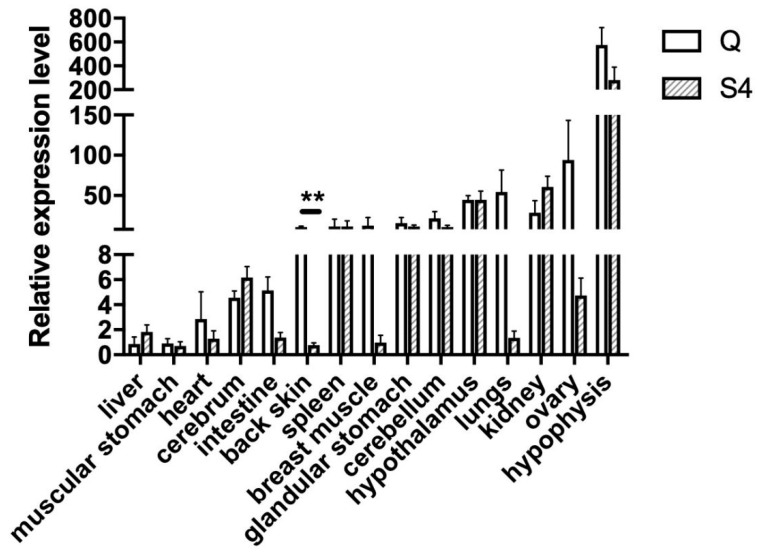
*BCO2* expression level in different chicken tissues in the Qingjiao Ma (Q) and Guangxi Huang (S4) chicken breeds. Relative expression analyses in 15 tissue samples of *BCO2* gene were normalized to the geometric average of *ACTB* (β-actin) and *GAPDH* genes. Error bars represent the SE, and ** indicates significant differences between different breeds for the same tissue (*p* ≤ 0.01).

*BCO2* was expressed in all tissues of Qingjiao Ma chickens. *BCO2* had a lower expression level in the heart, intestine, back skin, breast muscle, lungs and ovary in the Guangxi Huang (S4) compared with those in Qingjiao Ma (Q). However, only in the back skin was the average relative abundance of *BCO2* mRNA of the Guangxi Huang chickens significantly lower than that of the Qingjiao Ma (*p* < 0.01). The expression levels of *BCO2* were much higher in the hypophysis in both breeds compared with those in the other tissues (Figure 2).

**Figure 3:**
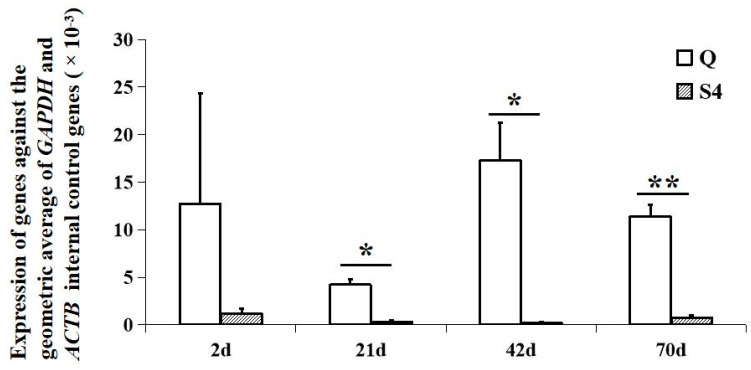
Expression of *BCO2* in chicken back skin at four different times. Relative expression analyses in back skin of *BCO2* gene were normalized to the geometric average of *ACTB* (β-actin) and *GAPDH* genes. Error bars represent the SE; * and ** indicate significant differences at *p* ≤ 0.05 and *p* ≤ 0.01, respectively, in gene expression between the Qingjiao Ma (Q) and Guangxi Huang (S4) chicken breeds.

As shown in Figure 3, *BCO2* was expressed in the back skin of Qingjiao Ma chickens at the age of 2, 21, 42, and 70 days, but the mRNA expression was very low, only slightly greater than zero, in the Guangxi Huang chickens. The mRNA expression of *BCO2* in the back skin of Qingjiao Ma chickens was significantly higher than that in Guangxi Huang chickens at the age of 21, 42, and 70 days (*p* < 0.05).

### 3.2. Single Nucleotide Polymorphism Scanning in BCO2 Coding Sequence 

**Figure 4:**
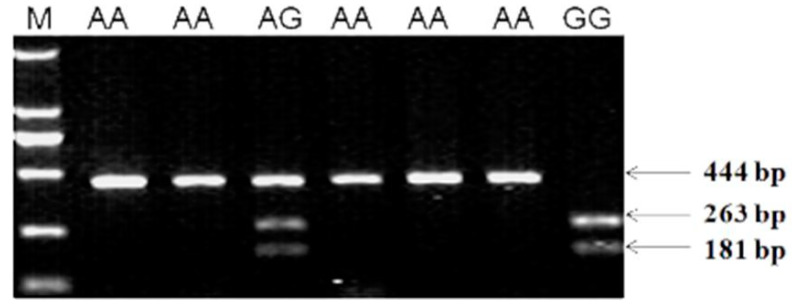
Agarose gel electrophoresis result of PCR-RFLP. The electrophoresis patterns obtained after digestion with *Sdu*I endonuclease at the c.890A>G locus. Agarose gel electrophoresis (1.5%) image showing the DNA restriction fragment patterns indicative of polymorphisms in *BCO2* gene after amplification with the primer pair YSD-F/R. The genotypes are shown at the top of the lanes, M, Marker DL2000 DNA Ladder (TaKara).

Scanning for SNPs in the *BCO2* CDS gene by TA-cloning and sequencing did not lead to previously described SNPs for this gene. However, a G/A mutation was found in exon 6 (c.890A>G, GenBank accession No. XM_417929.6). The 444 bp PCR amplicon containing the SNP was detected by digestion with *Sdu*I, resulting in allele G (263 + 181 bp) and allele A (444 bp) (Figure 4). The Qingjiao Ma breed had two genotypes, i.e., AA and AG, whereas the Guangxi Huang breed was only homozygous for the GG genotype. 

We also tested three SNPs, namely G>A, A>G, and G>A (chr24: 6,264,085 bp, 6,273,428 bp, and 6,287,900 bp, WUGSC 2.1/galGal3), in five Chinese indigenous chicken breeds; the results showed that some breeds carried alleles at this locus that are not so closely related to white-skin or yellow-skin alleles in our domestic breeds, such as Silkies, Fast-Growing Lingnanhuang Line A, and Guangxi Huang chickens. In contrast, the SNP c.890A>G screen showed that all five tested breeds carried alleles at this locus that are closely related to white-skin or yellow-skin alleles in Chinese domestic chicken (Table 2). This result was confirmed by PCR-RFLP in other Chinese indigenous chicken breeds (Table 3). The allelic frequencies of the SNP c.890A>G in white-skin and yellow-skin chicken breeds were notably different. As shown in Table 3, allele A was predominant in those breeds with a white skin, such as Qingjiao Ma, Youxi Ma, and Mahuang with black shank. Allele G was predominant in yellow skin breeds, including Huiyang Beard, Fast-Growing Lingnanhuang Line A, Mahuang with navy shank, and Guangxi Huang Chicken.

## 4. Discussion

Carotenoids are an important factor for the growth and health of birds and their color, and therefore carotenoid additives and vitamin A supplements may be added to poultry feed formulations. As an alternative for this, some studies have used established high-carotenoid maize for these supplements in laying hens and broilers [26,27,28]. Our results show that carotenoids are deposited in the skin of Guangxi Huang chicken with yellow skin, while in white-skin chickens, there is a lower deposition of carotenoids, as no β-carotene was detected, while the lutein and zeaxanthin were detected to be significantly lower in Qingjiao Ma chickens than that of yellow-skin chickens. In chickens, the *BCO2* gene is a classic yellow-color gene, and in line with these observations, there was almost no expression of the *BCO2* gene in the skin of the Guangxi Huang (yellow skin) breed, in contrast to the Qingjiao Ma (white skin) breed. The absence of *BCO2* expression in the skin of the Guangxi Huang breed was specific for the skin, as no significant differences in *BCO2* gene expression were observed among the two breeds in any of the other tissues investigated. Because BCO2 cleaves orange/yellow carotenoids into colorless apocarotenoids, it is concluded that *BCO2* is the gene that regulates the deposition of carotenoids in chicken skin. However, adding more color additives to poultry diets does not induce a change from white skin to yellow skin color, indicating that the coloring of the chicken skin is more complex. Furthermore, the presence of zeaxanthin in the feed might interfere with the absorption of b-carotene, as chickens fed on diets with low levels of zeaxanthin accumulated higher levels of retinol in the liver [29].

Scanning for the presence of skin color related SNPs (G>A chr24: 6,264,085 bp, A>G chr24: 6,273,428 bp and G>A chr24: 6,287,900 bp, WUGSC 2.1/galGal3) between yellow-skin and white-skin chicken did not lead to the identification of previously reported SNPs [15]. An explanation for this discrepancy may be related to differences in the chicken breeds used in the two studies. Instead, we found a new SNP in the *BCO2* gene, which, when tested in other Chinese indigenous chicken breeds, may be used as a genetic marker associated with skin color. The G/A mutation found in exon 6 of *BCO2* (c.890A>G, GenBank accession No. XM_417929.6) was a same-sense mutation and did not directly change the amino acids of protein, but they were not entirely negligible either, as they affected *BCO2* gene expression. Silkie breed displaying Fibromelanosis (FM), which is characterized by intense pigmentation of the dermal layer of skin across the entire body, results in a dark blue appearance when viewed through the clear epidermis [30], and when there are carotenoids depositing in the epidermis, this results in a yellow-to-dark-green appearance. Yellow pigmentation in the epidermis is determined by the W locus, at which the W allele inhibits epidermal xanthophyll pigmentation and is completely dominant to w [31,32]. We found 9 out of 30 birds have yellow-to-dark-green shank color and are different with other dark blue shanks, and the results of the SNP c.890A>G polymorphisms were consistent with this phenotype. However, although the *BCO2* may determine whether the skin is yellow or white, it cannot determine the yellowness value of skin [33].

Several reports indicate that copy-number variations are highly associated with specific phenotypes. For example, CNVs in intron 1 of the *SOX5* gene cause the Pea-comb phenotype in chickens [34], while two duplications containing *EDN3* result in an abnormal skin pigmentation phenotype in the silkie breed [30]. CNVs in the testis-specific Y-encoded protein (*TSPY*) gene have been linked to prostate cancer and human male infertility [35,36,37]. CNVs in the stem cell factor receptor (*KIT*) gene are linked to porcine white coat color [38], while CNVs in the agouti signaling protein (*ASIP*) gene influence the coat color of goat breeds [39]. We also detected the CNVs in *BOC2*, with the copy number in the Qingjiao Ma (white skin chicken) being almost two-fold larger than in the Guangxi Huang (yellow skin chicken) breed, suggesting that a lower CNV may lead to the downregulation of *BCO2* expression. However, this only indicated the difference in CNVs in the *BCO2* gene based on the TaqMan real-time PCR; we would give the information of gene structure with these CNVs by the re-sequencing and validation. 

Despite our observations, one has to keep in mind that SNPs may not be the only cause of differences in *BCO2* gene expression between the Qingjiao Ma and Guangxi Huang chicken breeds. There may be other factors that can be responsible for the reduced expression of the *BCO2* gene expression in the Guangxi Huang chicken breed, such as DNA methylation [40,41,42], histone modification [43,44,45], and UTR function and, thus, translational modification [46]. Additional experiments need to be performed to elucidate the specific difference in *BCO2* expression in the skin of Qingjiao Ma and Guangxi Huang chicken breeds without differences in gene expression in any other tissue. 

## 5. Conclusions

In conclusion, the reduced expression of the *BCO2* gene may be responsible for the deposition of carotenoids and, as a consequence, a yellow skin color in Guangxi Huang chickens. One SNP c.890A>G was found to be associated with the chicken skin color and may possibly, in the future, be used as a genetic marker in breeding for yellow skin in Chinese indigenous chickens.

## Figures and Tables

**Table 1:** The information of the used primers in the study.

Name	Sequence (5′-3′)	Annealing Temperature (°C)	Product Length (bp)
YSD-F	TCCTCTGATTGCTTTACTGACTTG	60	444
YSD-R	GGGAAGGAGGTATCATTGGAGA		
BCO2-F	ACTGGACCAAGTTTGTTGCCGT	58	192
BCO2-R	GTTGGAGCAATGGAGCATAGCA		
GAPDH-F	GGTGAAAGTCGGAGTCAACGG	59	108
GAPDH-R	TCGATGAAGGGATCATTGATGGC		
ACTB-F	CCCCAAAGCCAACAGAGAGA	59	158
ACTB-R	GGTGGTGAAGCTGTAGCCTCTC		
BCO2-A-F	CAGGGGAGATCACAACGGAC	56	352
BCO2-A-R	ATGAGCACCGGGAACCATTT		
BCO2-B-F	CACAGCTCCCGTCAAAGCTA	56	403
BCO2-B-R	ATGTTGGCATGAGCTCGTCA		
BCO2-C-F	CCGCATCTAGCAGAGCGATA	56	449
BCO2-C-R	AGTCCCAAAGTTTGTGCAGC		

**Table 2:** Genotype distribution for the SNP A, SNP B, SNP C, and SNP c.890A>G in the *BCO2* gene in 5 chicken breeds.

Breed	No. of Birds	SNP A ^1^			SNP B ^1^			SNP C ^1^			SNP c.890A>G		
		Genotype Distribution			Genotype Distribution			Genotype Distribution			Genotype Distribution		
		AA	AG	GG	AA	AG	GG	AA	AG	GG	AA	AG	GG
Silkies ^2^	30	1	9	17	15	9	1	24	0	0	17	0	9
Fast-Growing Lingnanhuang Line A	30	25	5	0	0	0	30	23	4	0	0	0	29
Guangxi Huang	30	28	1	0	0	9	21	30	0	0	0	0	30
Qingyuan Ma	30	30	0	0	0	0	30	30	0	0	0	0	30
Huiyang Beard	30	30	0	0	0	0	30	30	0	0	0	0	30

^1^ SNP A = nucleotide position 6,264,085, G/**A**; SNP B = nucleotide position 6,273,428, A/**G**; SNP C = nucleotide position 6,287,900, G/**A**; bold, underlined nucleotides are those associated with the yellow skin haplotype [12]. ^2^ Silkies is an old world-famous Chinese indigenous breed. It is featured with a number of special external traits, such as a crest, rose comb, black skin, green earlobe, muffs and beard, silky feathers, feathered shank, and polydactyly phenotypes [18].

**Table 3:** Allelic frequencies for the SNP c.890A>G in the *BCO2* gene in 7 chicken breeds.

Breed ^1^	No. of Birds	Genotypic Frequency			Allelic Frequency	
		AA	AG	GG	A	G
Qingjiao Ma	60	60	0	0	1.000	0.000
Guangxi Huang	60	0	0	60	0.000	1.000
Youxi Ma	44	29	15	0	0.830	0.170
Huiyang Beard	60	0	0	60	0.000	1.000
Fast-Growing Lingnanhuang Line A	60	0	0	60	0.000	1.000
Mahuang with black shank	78	74	4	0	0.974	0.026
Mahuang with navy shank	52	0	0	52	0.000	1.000

^1^ Yellow-skin chicken breeds: Huiyang Beard, Fast-Growing Lingnanhuang Line A, Mahuang with navy shank, and Guangxi Huang Chicken. White-skin chicken breeds: Qingjiao Ma, Youxi Ma, and Mahuang with black shank Chicken.
